# Stacking-induced fluorescence increase reveals allosteric interactions through DNA

**DOI:** 10.1093/nar/gky887

**Published:** 2018-10-02

**Authors:** Michael J Morten, Sergio G Lopez, I Emilie Steinmark, Aidan Rafferty, Steven W Magennis

**Affiliations:** School of Chemistry, WestCHEM, University of Glasgow, Joseph Black Building, University Avenue, Glasgow G12 8QQ, UK

## Abstract

From gene expression to nanotechnology, understanding and controlling DNA requires a detailed knowledge of its higher order structure and dynamics. Here we take advantage of the environment-sensitive photoisomerization of cyanine dyes to probe local and global changes in DNA structure. We report that a covalently attached Cy3 dye undergoes strong enhancement of fluorescence intensity and lifetime when stacked in a nick, gap or overhang region in duplex DNA. This is used to probe hybridization dynamics of a DNA hairpin down to the single-molecule level. We also show that varying the position of a single abasic site up to 20 base pairs away modulates the dye–DNA interaction, indicative of through-backbone allosteric interactions. The phenomenon of stacking-induced fluorescence increase (SIFI) should find widespread use in the study of the structure, dynamics and reactivity of nucleic acids.

## INTRODUCTION

**Figure 1.**
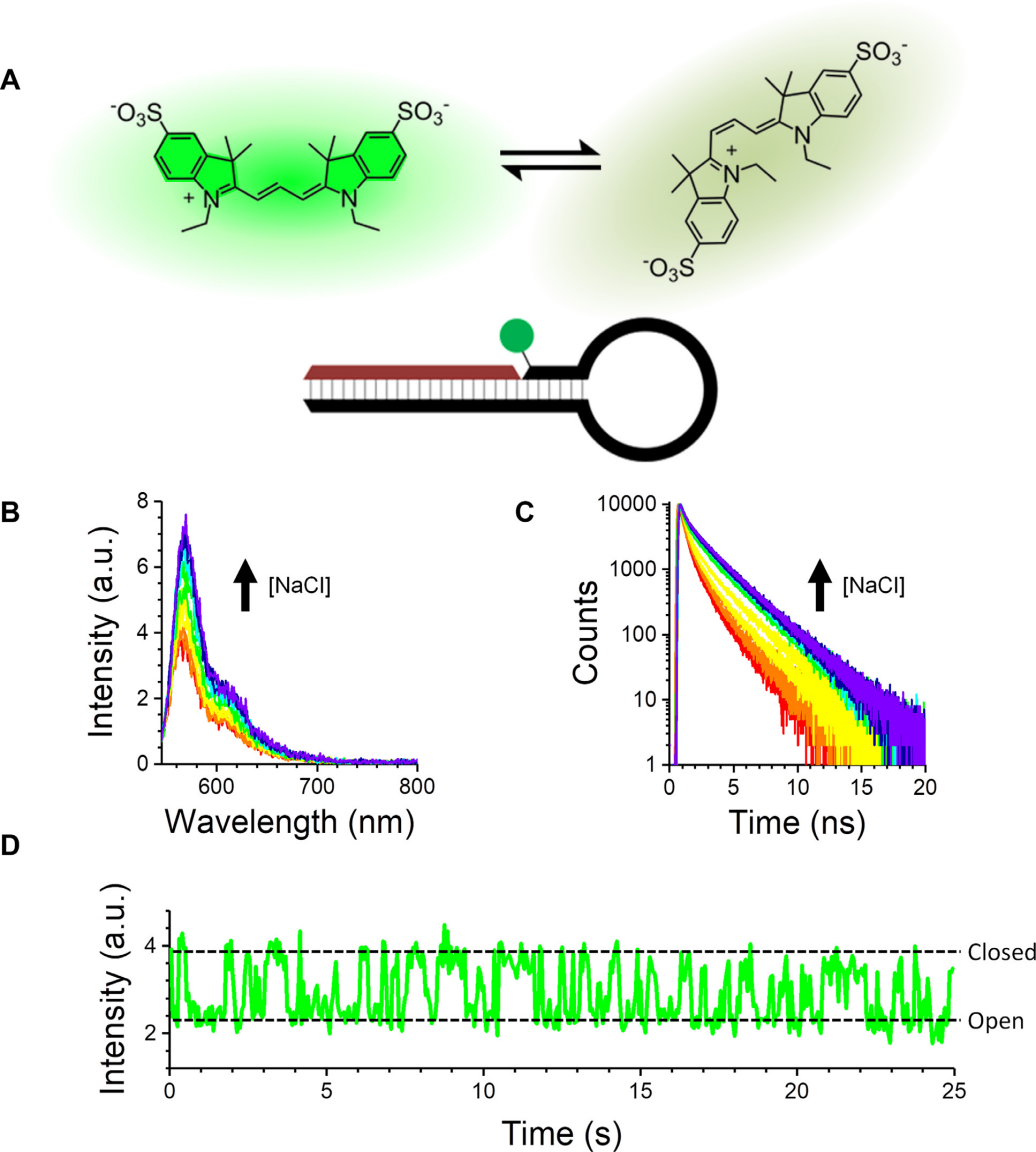
Fluorescence enhancement of Cy3 upon closing of a DNA hairpin. (**A**) Photoexcited Cy3 is known to isomerize between a bright *trans* isomer and a dark *cis* isomer. The schematic shows a nicked hairpin structure labelled on 3′ deoxythymidine (dT) with Cy3. (**B** and **C**) Addition of NaCl (0–460 mM) results in an increase of Cy3 emission intensity (B) and lifetime (C). (**D**) Representative single-molecule time trace shows two-state dynamics (the buffer contained 50 mM NaCl).

The environmental sensitivity of cyanine dyes such as Cy3 in the vicinity of a protein, known as protein-induced fluorescence enhancement (PIFE), is a powerful approach to studying DNA–protein interactions (1,2). The enhancement results from changes in the rate of photoisomerization of the cyanine dye, and the mechanism for this is now well established (3). Upon excitation, Cy3 in the *trans* form is highly emissive, but it can also photoisomerize from the excited *trans* state to the ground state of the *cis* isomer (Figure 1A) (4). The more sterically hindered the rotation of the double bond is, the longer Cy3 spends in the *trans* conformation, resulting in an increased emission quantum yield (5). This allows the dye to report on its microenvironment. Here we introduce a new strategy for enhancing the fluorescence of Cy3 through the site-specific stacking of Cy3 in a nick, gap or overhang in duplex DNA. Using only a single Cy3 label, we are able to probe multiple aspects of DNA structure and dynamics. First, we monitored the opening and closing of a DNA hairpin, measuring the dynamics down to the single-molecule level. We then took advantage of the dye’s sensitivity to local DNA structure, revealing long-range perturbations due to the presence of a single abasic site up to 20 base pairs (bp) away from the site of dye stacking; we discuss this finding in the context of the recent report of allostery in DNA–protein interactions (6).

## MATERIALS AND METHODS

### Materials

NaCl (analytical reagent grade) was purchased from Fisher Scientific (Loughborough, UK). TRIS base (BioUltra for luminescence, ≥99.8%) and TRIS hydrochloride (reagent grade, ≥99%) were purchased from Sigma Life Sciences (Dorset, UK). All of the chemicals were used as received from the manufacturers.

The DNA sequences were synthesized and purified by Purimex GmbH [Grebenstein, Germany; triple high performance liquid chromatography (HPLC) and polyacrylamide gel electrophoresis (PAGE), supplied in water; sequences a–c in Supplementary Data] or IBA GmbH (Göttingen, Germany; double HPLC and PAGE, supplied lyophilized; sequences d–e in Supplementary Data). The DNA sequences were dissolved in a buffer containing 20 mM Tris and 50 mM NaCl (pH 7.8) to obtain 100 μM DNA stock solutions. These solutions were mixed in a 1:2 (labelled hairpin:complementary sequence) ratio and annealed by placing them in a water bath at 90°C that was then allowed to cool overnight. The DNA solutions were stored at −20°C.

All ensemble experiments were made in 20 mM Tris–HCl (pH 7.8) and at various NaCl concentrations. Single-molecule experiments used the same buffer with the addition of 6% glucose (w/v), glucose oxidase, catalase and Trolox. All measurements were made at 21 ± 1°C.

### Absorption spectroscopy

The UV-visible spectra were recorded using a Cary 60 UV-Vis spectrometer (Agilent Technologies, Santa Clara, USA). The DNA concentrations were calculated from the DNA absorbance at 260 nm using the molar extinction coefficients provided by the manufacturer. The dye concentrations were calculated using the molar absorption coefficient of Cy3 (*ϵ* = 150 000 M^−1^ cm^−1^ at 550 nm) (7).

### Fluorescence spectroscopy

The fluorescence spectra and fluorescence decays were recorded using a FluoTime 300 fluorescence spectrometer (PicoQuant, Berlin, Germany). The excitation light was provided by a Fianium WhiteLase super-continuum laser (NKT Photonics, Birkerød, Denmark) and passed through a SuperChrome filter (NKT Photonics, Birkerød, Denmark) before arriving at the sample holder. The full width at half maximum (FWHM) of the instrument response function was ≈120 ps at 10 MHz rep rate. The excitation and emission wavelengths were 532 and 565 nm, respectively. The excitation bandpass was 10 nm, while the emission bandpass was 2.7 nm. All of the fluorescence measurements were carried out under magic-angle conditions to avoid fluorescence polarization artefacts. The time channel width was 8 ps. In all cases, the absorbance of Cy3 was ≤0.1 to avoid inner-filter effects. All of the measurements were recorded until they reached ten or twenty thousand counts in the peak channel. The fluorescence decays were best fit to either bi- or tri-exponential decay functions. The fitting was carried out using the FluoFit data analysis software (PicoQuant, Berlin, Germany). Only those fits that resulted in *χ*^2^ values close to 1 and whose residuals were randomly distributed around zero were deemed acceptable.

Two different average lifetimes (the amplitude-weighted average lifetime, $\langle \tau \rangle$, and the intensity-weighted average lifetime, $\bar{\tau }$) were calculated as follows:

\begin{equation*}\left\langle \tau \right\rangle {\rm{\ }} = \frac{{\mathop \sum \nolimits_{i = 1}^n {\alpha _i}{\tau _i}}}{{\mathop \sum \nolimits_{i = 1}^n {\alpha _i}}}\ \end{equation*}

\begin{equation*}\bar{\tau }{\rm{\ }} = \frac{{\mathop \sum \nolimits_{i = 1}^n {\alpha _i}{\tau _i}^2}}{{\mathop \sum \nolimits_{i = 1}^n {\alpha _i}{\tau _i}}},\ \end{equation*}

where ${\alpha _i}$ and ${\tau _i}$ are the fractional amplitudes and lifetimes of the *i*th decay component, respectively.

### Single-molecule TIRF microscopy

Single-molecule total internal reflection fluorescence (TIRF) measurements were made and analysed as reported previously (8).

### AV calculations

Accessible volume (AV) calculations were performed using the Förster resonance energy transfer (FRET) positioning and screening (FPS) software described by Kalinin *et al.* (9). Briefly, the duplex structures were modelled as B-form DNA helices, with two nucleotides deleted from the model to create a gap. The parameters for a sulfo-Cy3-NHS ester dye (long linker) were taken directly from the procedure written by Kalinin *et al.*; the C5 of the pyrimidine ring was used as the connecting atom of the labelled deoxythymidine (dT) in the duplex structures. The resulting structures were visualized using Pymol.

## RESULTS

### Stacking-induced fluorescence increase

We first designed a fully complementary DNA hairpin labelled with the cyanine dye Cy3 attached to the 3′ end of the stem region (Figure 1A and Supplementary Figure S1) (8,10). Cy3 is usually attached in either the neutral form or negatively charged sulfonated form to the 3′ or 5′ end of an oligonucleotide, or it is incorporated internally by direct attachment to a base; various linkers have been used for covalent attachment (4). We adopted a hybrid approach, whereby a sulfonated Cy3 dye was attached to dT at the 3′ end, rather than internally (Supplementary Figure S1); we hypothesized that this labelling strategy would not impede hairpin closing. The hairpin loop is a 32 nucleotide polyA, and the stem is a GC-rich sequence of six nucleotides. The oligonucleotide extends in the 5′ direction to incorporate a 20-nucleotide handle with the 5′ terminal nucleotide modified to include a biotin moiety at the end of a carbon linker. This handle allows the hairpin to be immobilized for single-molecule experiments.

We used steady-state fluorescence spectroscopy, time-correlated single-photon counting (TCSPC) and TIRF microscopy to monitor the Cy3 emission. Upon addition of NaCl to an aqueous solution of the hairpin, we observed a large increase in ensemble fluorescence intensity (Figure 1B) and lifetime (Figure 1C). Single-molecule traces showed that the dynamics followed two-state kinetics, which agreed with the salt-induced opening and closing of similar hairpins observed previously using FRET (Figure 1D) (8,10). We hypothesized that the increase was due to restricted photoisomerization of Cy3 (4), though it was not clear what interaction was causing such a well-defined enhancement.

**Figure 2.**
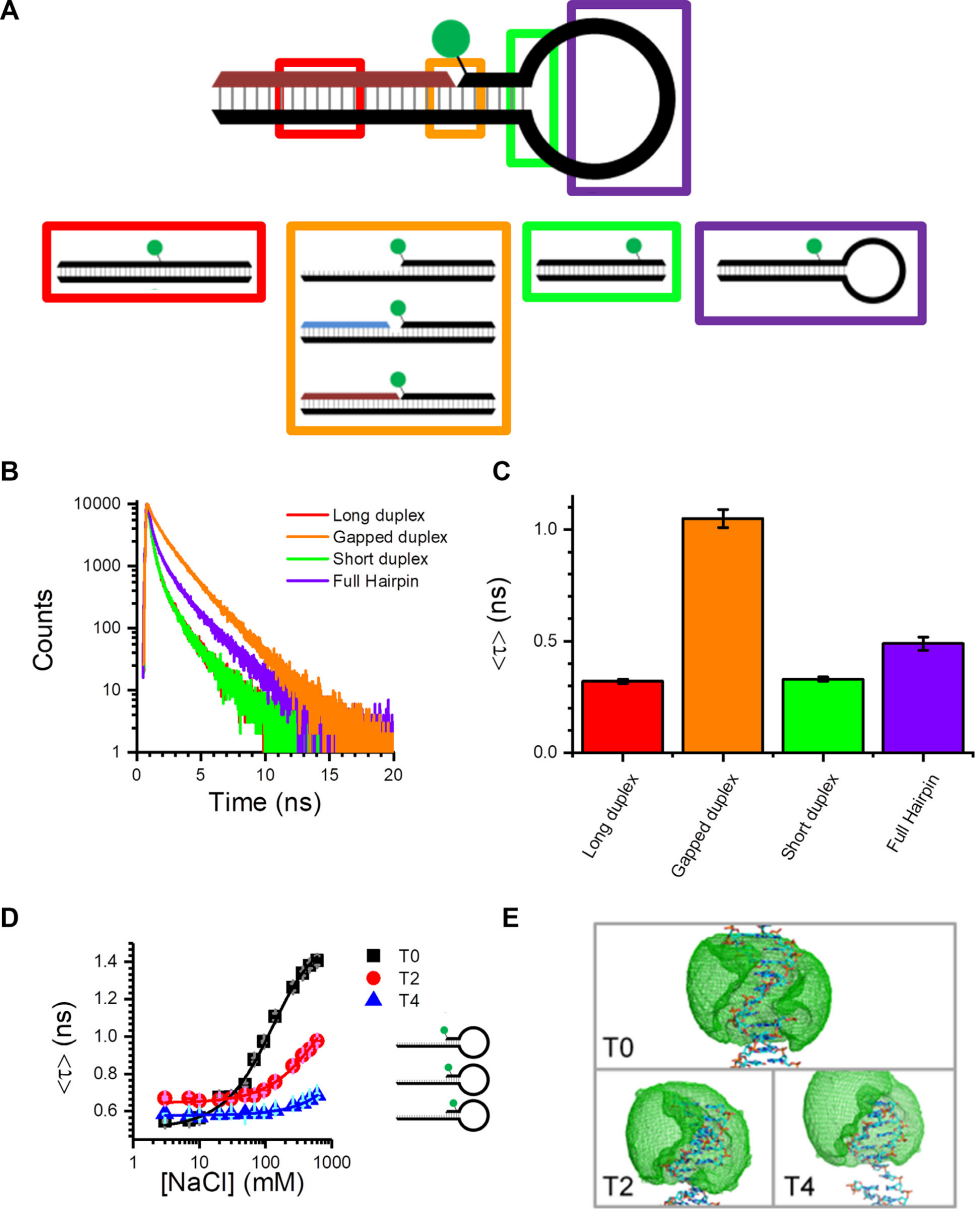
Stacking-induced fluorescence increase of Cy3. (**A**) Immobile DNA structures designed to probe the possible interactions between the Cy3 dye and the DNA hairpin (see Supplementary Figure S2 for additional structures). (**B** and **C**) Time-resolved decays (B) and amplitude-weighted average lifetimes (C) for Cy3 attached to representative duplex structures (the buffer contained 50 mM NaCl). (**D**) Amplitude-weighted average lifetime as a function of NaCl concentration for hairpins with different dye positions. Moving the dye away from the ssDNA–dsDNA junction reduces the enhancement. (**E**) AV calculations illustrate that the dye can explore up to four bases along duplex DNA (see Supplementary Figure S7 for expanded versions of these). Error bars in (C) and (D) represent the standard deviation (*N* = 3).

The nicked hairpin in Figure 1A has a number of distinct structural features, some of which depend on the conformational state of the hairpin. Therefore, we designed a series of DNA structures to uncover the origin of the fluorescence increase for Cy3 (Figure 2A and D; Supplementary Figure S2). We tested for the influence of the hairpin loop, the proximity of the dye to the end of duplex DNA, the effect of local attachment to single-stranded DNA (ssDNA) versus double-stranded DNA (dsDNA), and the proximity of the Cy3 to a nearby nick, gap or single-strand overhang.

Before studying the Cy3 structures, we replaced the Cy3 with Cy3B, which is an analogue of Cy3 that does not undergo isomerization (4). Upon addition of NaCl to a Cy3B-containing hairpin, there was no change in the fluorescence intensity or lifetime, or fluctuations in single-molecule intensity (Supplementary Figure S3 and Supplementary Table S1). This supported the hypothesis that Cy3 emission enhancement in hairpins was due to restricted photoisomerization. We then focused on Cy3-labelled structures.

We found that time-resolved emission from Cy3 was particularly sensitive to the dye’s local environment. In all TCSPC measurements of Cy3, the decays were multiexponential (e.g. Figure 2B), requiring three decay components (see Supplementary Tables S1 and S2 for representative TCSPC data recorded in the presence of 50 mM NaCl). The fitted lifetimes agree with previous measurements of Cy3 (5). For example in the presence of 50 mM NaCl, the longest decay component was ∼2–2.4 ns, which is assigned to a predominately stacked *trans* conformation, while the short component at ∼200–300 ps is assigned to unstacked Cy3 due to its similarity to free Cy3 (5). The intermediate lifetime for the duplexes and hairpins was ∼0.6–1 ns; we attribute this component to another well-defined conformation such as minor groove binding, which is known to occur for cyanine molecules (11). Since changes to the fraction of dyes undergoing non-radiative photoisomerization will lead to changes in both emission intensity and lifetime, we also found that the average decay time was a particularly useful diagnostic of the Cy3 environment (e.g. Figure 2C). These three lifetimes may reflect three distinct dye microenvironments or they may be due to distributions around those lifetimes. Furthermore, at an NaCl concentration of 50 mM, there will be a contribution from Cy3 in the open hairpin conformation. As the lifetimes for Cy3 on ssDNA are similar to those on dsDNA, we cannot easily distinguish between open and closed conformations (Supplementary Table S2). However, as we will see below, we have also studied the hairpins at high salt concentrations, where the closed conformation predominates.

We first examined a series of dsDNA structures in the absence of a single-stranded loop. When Cy3 was attached internally to dsDNA (red box in Figure 2A), there was a large reduction in lifetime in comparison with the nicked hairpin (Figure 2B and C; Supplementary Figure S4 and Supplementary Table S2). Using a shorter duplex, where the distance from the dye to the end of the duplex (6 bp) was equivalent to the distance from the dye to the hairpin loop (green box in Figure 2A), produced similar results (Figure 2B and C; Supplementary Figure S4 and Supplementary Table S2). There was also little effect of NaCl (Supplementary Figures S4 and S5), in contrast to the hairpin, and only stable single-molecule traces were observed for short and long duplexes (Supplementary Figure S6). In addition, when Cy3 was end-labelled on ssDNA, the decay was similar to when internally labelled on a fully complementary duplex (Supplementary Table S2). In contrast, a large change in intensity/lifetime occurred (Figure 2B and C; Supplementary Table S2) whenever the dye was in a duplex with the dye adjacent to either a nick, gap or single-strand overhang (orange box in Figure 2A). Once again, the duplex structures exhibited little salt dependence (Supplementary Figure S5) and showed no intensity fluctuations (Supplementary Figure S6). This implied that proximity to an ssDNA–dsDNA junction is important for the enhancement, but not the dynamics or dependence on NaCl. Furthermore, a stable hairpin loop structure that cannot undergo opening and closing at room temperature (purple box in Figure 2A) showed only a small increase in fluorescence intensity and lifetime, relative to the intact duplexes (Figure 2B and C; Supplementary Table S1), and did not display any dynamics at the single-molecule level (Supplementary Figure S6). This showed that interaction with the hairpin loop was not responsible for the fluorescence enhancement or the two-state intensity fluctuations.

We also designed hairpin structures in which the dye was located at different distances from the ssDNA–dsDNA junction; these are labelled T0, T2 and T4 according to the number of bases distant from the junction (Figure 2D and E). These three hairpins all have similar sequence contexts in the stem region; the main difference is that the T2 junction has a larger loop, two extra base pairs and the unpaired base at the overhang is G instead of T. As shown by TCSPC measurements, the enhancement was progressively lower as the dye was moved away from the junction (Figure 2D). This agreed with AV calculations (9), which showed that for T4, the dye is very unlikely to stack at the overhang (Figure 2E and Supplementary Figure S7). Single-molecule measurements also displayed a reduction in enhancement as the dye was moved away from the junction (Supplementary Figure S6). To check if there was a dependence on the method of Cy3 conjugation, we substituted the Cy3-dT label for a 3′-Cy3 (Supplementary Figure S1). This produced a very similar enhancement for the T0 overhang structure (Supplementary Figure S8) and the nicked hairpin (Supplementary Figure S9). Therefore, it appears that the 3′-Cy3 linker is long enough to allow a similar interaction as for the dT attachment.

Overall, the data presented in this section demonstrate that Cy3 can stack in a nick, gap or overhang region of DNA, leading to fluorescence enhancement. Furthermore, we showed that single-molecule intensity fluctuations are due to salt-dependent hairpin dynamics rather than stacking dynamics; we investigated this aspect further, as detailed below.

### Single-molecule dynamics

Measurements on hairpins with either a nick, 2 bp gap, or single-strand overhang showed that the enhancement can be tailored by adjusting the salt concentration (Figure 1C). DNA and RNA hairpin dynamics are commonly probed using FRET, whereby the donor dye and acceptor dye/quencher are far apart in the open state (low FRET) and close together following hybridization (high FRET) (10,12–18). By studying single immobilized hairpins, the modulated FRET signal due to opening and closing can be measured, giving direct access to the underlying rate constants. As shown above (Figure 1D), we could measure hairpins labelled with only one Cy3 dye down to the single-molecule level.

**Figure 3.**
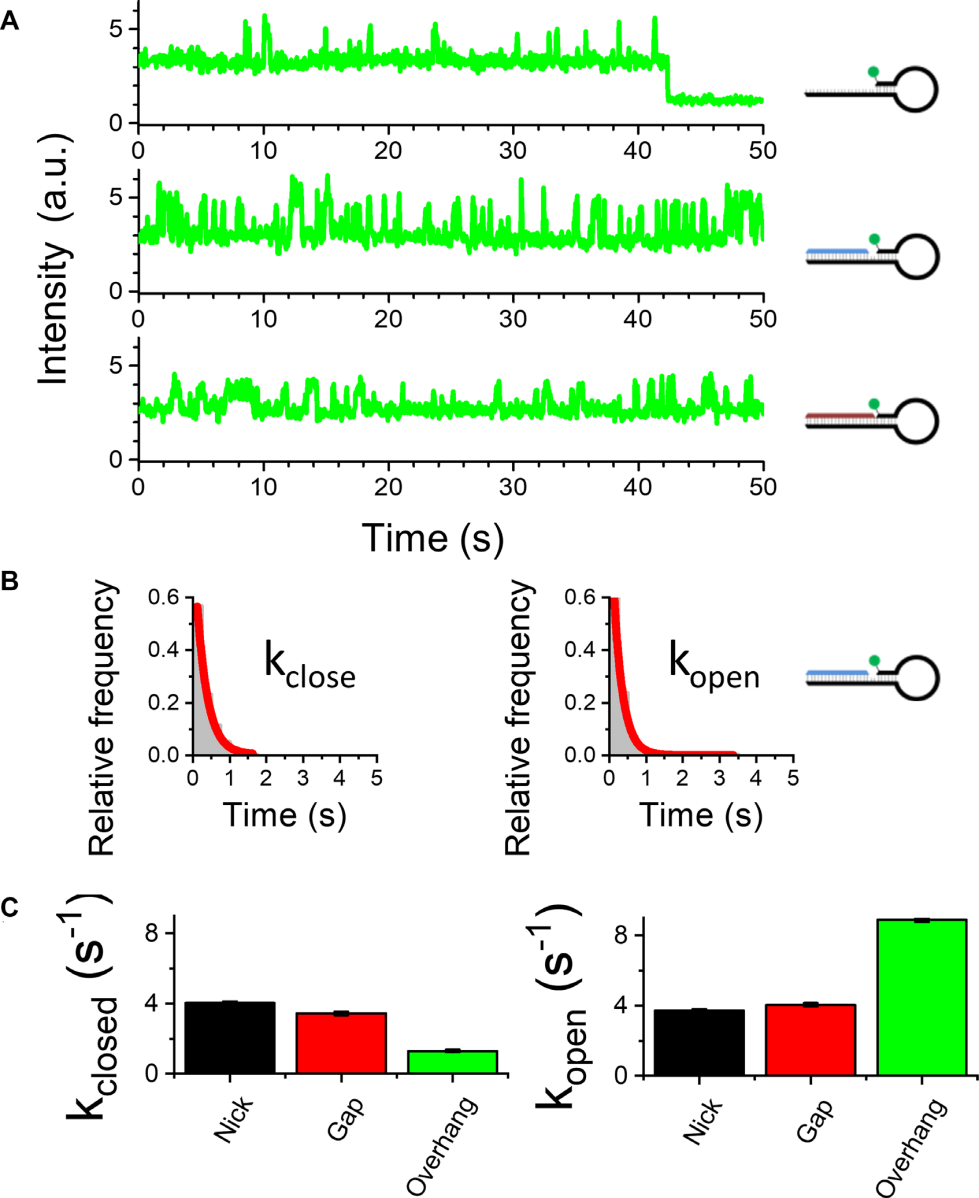
Monitoring single-molecule hybridization kinetics. (**A**) Representative single-molecule time traces for nicked, gap and overhang hairpin structures. (**B**) Dwell-time histograms can be fitted to a two-state model. (**C**) Opening and closing rates for the hairpins from the fits of the dwell-time histograms. The buffer contains 10 mM NaCl. Error bars in (C) represent the standard error for the single exponential fits. The number of open/closed transitions is >2000 from >100 molecules.

The single-molecule measurements showed that the hairpin exhibits two-state dynamics under all conditions (Figure 3A and B) and that the longer lifetime correlates with the increased on-time in the bright state. The transition from the open to closed state increases the fluorescence of the individual Cy3 dyes by a factor of up to ∼2.5, which is similar to the maximum enhancement seen by proteins binding to DNA (2). The two-state dynamics indicates that other processes such as Cy3 photoisomerization (with reported time constant of ∼10 μs (19)) and interconversion between different dye conformations (e.g. stacked versus unstacked) are much faster than the temporal resolution of the TIRF experiment (∼50 ms); therefore, the fluorescence intensity of Cy3 in either the open or closed conformation is the time-averaged fluorescence from all the bright and dark states that are present.

The dwell times in the two fluorescent states were used to calculate the opening and closing rates of individual hairpins (Figure 3C). For the nicked and gapped hairpins, the opening and closing rates (in the presence of 50 mM NaCl) are almost the same, whereas for the overhang, the opening rate is faster and closing rate slower. This suggests that the dye interaction with the nick or gap increases the stability of the closed conformation. Importantly, we have shown that the dynamics of hairpin opening and closing can be reliably monitored at the single-molecule level using only one dye.

### Dye stacking as a function of distance from an abasic site

**Figure 4.**
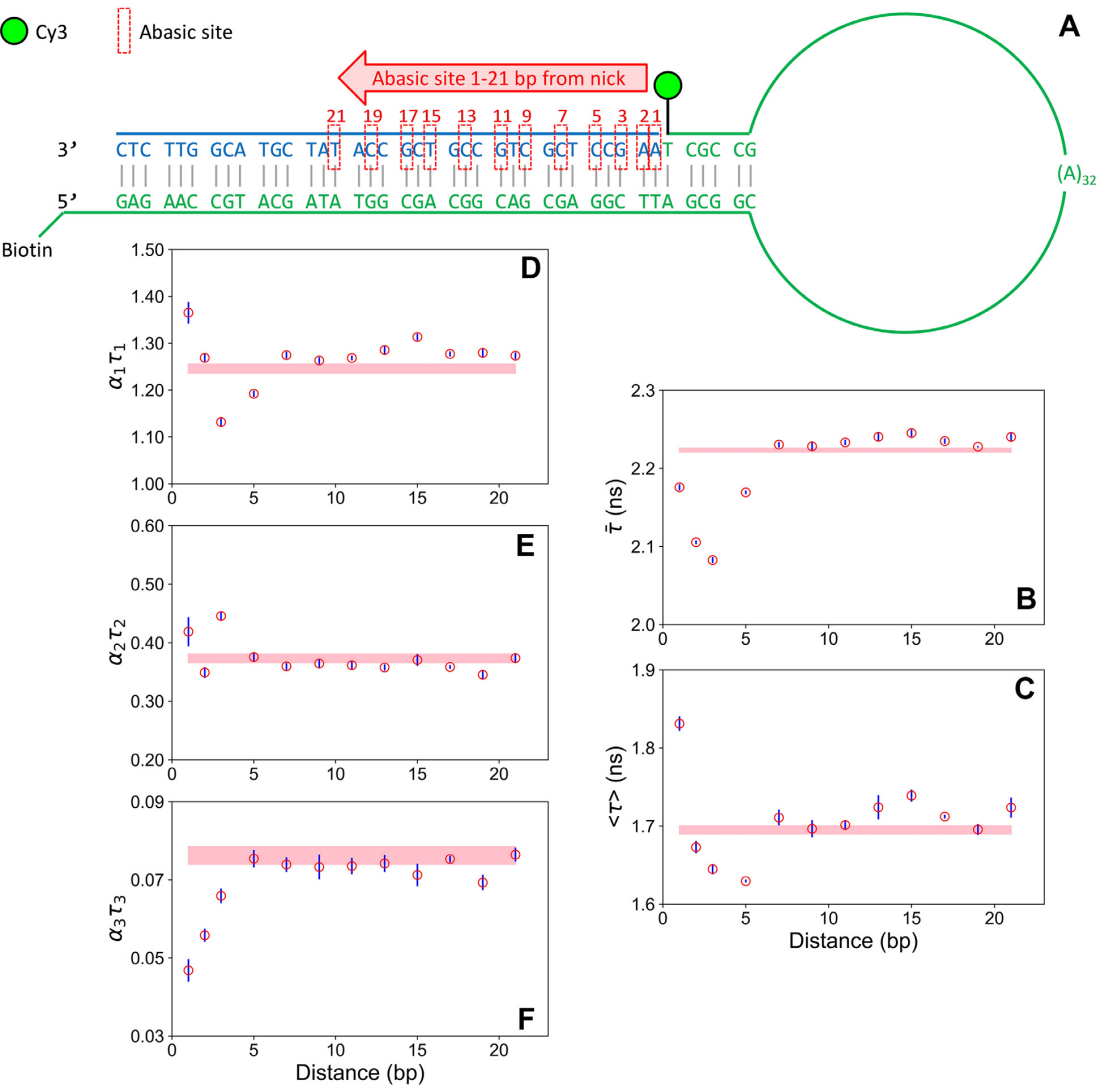
Effect of a remote abasic site on Cy3 stacking-induced fluorescence increase. (**A**) Schematic for hairpin structures with a single abasic site. (**B–F**) Time-resolved fluorescence as a function of the position of the abasic site. (B) Intensity-weighted average lifetime. (C) Amplitude-weighted average lifetime. (D–F) Fractional intensity (lifetime*amplitude) for the long (D), intermediate (E) and short (F) decay components. The error bars represent the standard error of the mean (three independent experiments with a total of thirteen decay measurements). The shaded bar (pink) represents the mean lifetime and standard error for the fully complementary control sequence (i.e. with no abasic site). The buffer contains 600 mM NaCl. See Supplementary Table S3 for fit parameters.

Having established that dye stacking can monitor hybridization, we next attempted to use the Cy3 fluorescence to probe changes to the duplex structure itself. We designed new hairpins that incorporated a single abasic site at different distances from a nick. We chose abasic modifications because they are one of the most common types of DNA lesion (20). They are also a well-defined structural perturbation that can be reliably incorporated via simple modifications to oligonucleotides. We used a nicked hairpin and sufficient NaCl (600 mM) to ensure the hairpin was always closed. The hairpin structures (Figure 4A) were very similar to the nicked hairpin used above; the only difference was the extended duplex region to increase the number of possible abasic sites. Only one abasic site was used per hairpin and this site was positioned either adjacent to the nick (defined here as 1 bp from the nick) or at 11 other positions up to 21 bp away. We also studied an identical fully complementary hairpin (i.e. with no abasic sites) as a control. We recorded time-resolved decays for each sample under identical conditions. As with the other hairpins (Figure 2), all of the decays were well described by three lifetime components (Supplementary Table S3) centred on 2.6–2.7 ns for the stacked conformation, 300 ps for unstacked Cy3 and an intermediate lifetime ∼1.3 ns due to an intermediate conformation. We note that half of the Cy3 dyes are now in the long-lifetime state, and that this lifetime is very similar to that of Cy3B (Supplementary Figure S3a), showing that photoisomerization is almost completely suppressed for these Cy3 dyes.

We first plotted the average lifetime of the fluorescence, using the extracted lifetimes and amplitudes (Figure 4B and C). We calculated both the intensity-weighted average lifetime (which is the average time in the excited state) and the amplitude-weighted average lifetime (which is proportional to the steady-state intensity). Both plots are qualitatively similar, showing a sharp decrease in average lifetime for the first few abasic sites, relative to the baseline average for the fully complementary structure; we note that the amplitude-weighted average lifetime is longer for the first abasic position, which we attribute to direct stacking into the abasic site. The average lifetime then increases again until the structure with an abasic site 9 bp away has the same value as the control structure. Notably, the average lifetime then increases further, indicative of an increase in stacking, until it peaks at ∼15 bp, after which it decays again until by 19 bp it is back to the baseline. This modulation matches the 10 bp helical pitch of B-form dsDNA, which indicates that the origin is due to through-backbone perturbations.

The values of the three lifetimes vary slightly from sample to sample (Supplementary Figure S10 and Supplementary Table S3). As a result, the decays cannot be fitted globally across the whole dataset (i.e. using three lifetimes as global parameters and allowing the amplitudes to vary). Instead, we plotted the fractional intensity of each component (defined as the product of the lifetime and amplitude) to illustrate how each decay component varies with abasic position (Figure 4D–F). The fractional intensity represents the contribution that the dye conformation with that particular lifetime makes to the steady-state intensity (21). It is clear that the strong modulation seen in the average lifetimes (Figure 4B and C) is also apparent in the individual components. There is a strong anticorrelation in the short (unstacked) and long lifetime (stacked) components when the abasic site is close to the dye. This is likely due to a direct insertion of the dye into the abasic site, as discussed above. Notably, there is also anticorrelation close to 15 bp, matching the peak observed in the average lifetime plots. In contrast, the intermediate lifetime has a small peak around 3 bp from the nick and then is relatively constant. As shown already by AV (Figure 2E and Supplementary Figure S7), the dye and linker cannot explore more than a distance of around four bases away. Therefore, these experiments reveal that a remote abasic site can perturb the local dye interaction with the DNA backbone over a long range (∼2 helical turns), dependent upon both distance and relative base positioning.

## DISCUSSION

Taken together, the data for the various dsDNA and hairpin structures (Supplementary Figure S2) can be explained by an enhancement mechanism due to stacking of Cy3 in a nearby nick, gap or overhang. This site-specific enhancement, which we term stacking-induced fluorescence increase (SIFI), has not been reported before. One well-known interaction of Cy3 is the stacking of cyanine dyes on the end base pair of a DNA duplex (22,23), but none of the structures examined here feature a dye near the blunt duplex end. While we observe a fluorescence increase in going from the ssDNA to annealed hairpin, Levitus *et al.* showed previously that when sulfonated Cy3 is attached to a T on the 5′ end of ssDNA, the quantum yield decreases if the ssDNA is annealed to complementary strands of various lengths; this included a structure with an overhang that is similar in length, but with opposite polarity, to the one studied here (orange box in Figure 2A) (5). The 5′ linker used in the earlier study (5) was also much shorter than the one used here, possibly preventing stacking. This suggests that there may be an opportunity to further tune the enhancement by tailoring the linker and sequence.

The benchmark for fluorescence probing of DNA structure and dynamics is FRET, particularly when done at the single-molecule level (9,24,25). While there are many advantages to FRET, among the disadvantages are the requirements for two fluorescent dyes (or a dye and quencher) and the fact that the dyes are normally attached to a molecule of interest via a long tether, rendering the technique rather insensitive to local changes. Fluorescent analogues of nucleobases can offer local information (26,27), but these probes are not yet suitable for single-molecule analysis. The approach described herein has a rather unique combination of features. Like PIFE (2), it takes advantage of the enhancement of emission that results from a reduction in photoisomerization, but it can be used in the absence of protein (3). It requires only one dye, it can operate at the single-molecule level and it can probe local and global structural rearrangements.

In addition to monitoring hairpin dynamics, we show that the dye–DNA interaction is extremely sensitive to local changes near the site of stacking, as demonstrated by the ability to reveal long-range perturbations to the DNA backbone. Communication between remote sites is a hallmark of allostery, first proposed by Monod *et al.* in terms of conformational changes in proteins (28). There has been a great deal of interest recently regarding allosteric interactions in DNA–protein systems, notably the demonstration of ∼10 bp oscillations in protein-binding efficiency as a function of the location of a second bound protein (6). Theoretical work (29) and molecular dynamics (MD) simulations (6,30) supported a model of DNA deformation mediated through the backbone. Such interactions are important in the context of processes such as gene regulation (31–33). Kim *et al.* showed using MD simulations that spatial correlations between the major groove widths at two positions oscillate with 10 bp periodicity; similar modulation was observed experimentally for protein binding (6). In the context of the model of dynamic allostery, first proposed by Cooper and Dryden (34), this can be viewed as the binding of one protein affecting long-range vibrational modes, which then entropically favours or disfavours binding of another protein at a second site. We have shown remarkably similar modulated binding behaviour (i.e. Cy3 stacking) over long distance due only to the location of a single abasic site. This is direct evidence, in the absence of protein, of through-backbone communication in DNA. It will be of interest to use the SIFI approach described here to investigate other aspects of DNA structure and dynamics.

## DATA AVAILABILITY

The data that underpin this work are available at http://dx.doi.org/10.5525/gla.researchdata.668.

## Supplementary Material

Supplementary Data
