# Supplementary material for: Stacking-induced fluorescence increase reveals allosteric interactions through DNA
Source: Nucleic Acids Res. 2018 Oct 2;46(21):11618–26. doi: 10.1093/nar/gky887 (PMC6265455; doi:10.1093/nar/gky887)
Supplement: Supplementary Data [file gky887_supplemental_files.pdf]

Supplementary Information for:

Stacking-induced fluorescence increase reveals allosteric interactions through DNA

Michael J. Morten, Sergio G. Lopez, I. Emilie Steinmark, Aidan Rafferty and Steven W. Magennis*

School of Chemistry, WestCHEM, University of Glasgow, Joseph Black Building, University Avenue, Glasgow, G12 8QQ, UK,

Corresponding Author

*E-mail: steven.magennis@glasgow.ac.uk

The PDF includes:

Oligonucleotide Sequences

Supplementary Figures 1-10

Supplementary Tables 1-3

Oligonucleotide sequences

a) DNA hairpins

The sequences of the DNA constructs used are shown below, where X = dT-Cy3, unless otherwise stated.

Overhang (complementary regions are underlined)

T0

5'-/biotin/ TGG CGA CGG CAG CGA GGC TTA GCG GCA AAA AAA AAA AAA AAA AAA AAA AAA AAA AAA AGC CGC **X**

T2

5'-/biotin/ TGG CGA CGG CAG CGA GGC TTC GAG GCA AAA AAA AAA AAA AAA AAA AAA AAA AAA AAA AGC C**X**C G

T4

5'-/biotin/ TGG CGA CGG CAG CGA GGC TTG GCG ACA AAA AAA AAA AAA AAA AAA AAA AAA AAA AAA AG**X** CGC C

Nick

Top: 5'- AAG CCT CGC TGC CGT CGC CA

Bottom: 5'-/biotin/ TGG CGA CGG CAG CGA GGC TTA GCG GCA AAA AAA AAA AAA AAA AAA AAA AAA AAA AAA AGC CGC **X**

Gap

Top: 5'- GCC TCG CTG CCG TCG CCA

Bottom: 5'-/biotin/ TGG CGA CGG CAG CGA GGC TTA GCG GCA AAA AAA AAA AAA AAA AAA AAA AAA AAA AAA AGC CGC **X**

Fully paired

5'-/biotin/ TGG CGA CGG CAG CGA GGC TTA GCG GCA AAA AAA AAA AAA AAA AAA AAA AAA AAA AAA AGC CGC **X**AA GCC TCG CTG CCG TCG CCA

b) DNA Duplexes

Overhang

Top: 5'- CGC CTC TAT CCG TGC CGC **X**

Bottom: 5'- /biotin/ TGG CGA CGG CAG CGA GGC TTA GCG GCA CGG ATA GAG GCG

Nick

Top 1: 5'- CGC CTC TAT CCG TGC CGC **X**

Top 2: 5'- AAG CCT CGC TGC CGT CGC CA

Bottom: 5'- /biotin/ TGG CGA CGG CAG CGA GGC TTA GCG GCA CGG ATA GAG GCG

Gap

Top 1: 5'- CGC CTC TAT CCG TGC CGC **X**

Top 2: 5'- GCC TCG CTG CCG TCG CCA

Bottom: 5'- /biotin/ TGG CGA CGG CAG CGA GGC TTA GCG GCA CGG ATA GAG GCG

Fully paired

Long

Top: 5'- CGC CTC TAT CCG TGC CGC XAA GCC TCG CTG CCG TCG CCA

Bottom: 5'- /biotin/ TGG CGA CGG CAG CGA GGC TTA GCG GCA CGG ATA GAG GCG

Short

Top: 5'- GCC GC**X** AAG CCT CGC TGC CGT CGC CA

Bottom: 5'- /biotin/TGG CGA CGG CAG CGA GGC TTA GCG GC

c) Single-stranded DNA

5' - /biotin/TGG CGA CGG CAG CGA GGC TTT TTT TTT TTT TTT TTT TTT TTT **X**

d) Hairpins with abasic sites

The abasic site is denoted by (a).

Sequence d1.

5'-2 GAG AAC CGT ACG ATA TGG CGA CGG CAG CGA GGC TTA GCG GCA $(A)_{30}$

AGC CGC3

Where 2 = Biotin hexyl

3 = dT-Cy3

Sequence d2.

5'-AA GCC TCG CTG CCG TCG CCA TAT CGT ACG GTT CTC

Sequence d3.

5'-(a)A GCC TCG CTG CCG TCG CCA TAT CGT ACG GTT CTC

Sequence d4.

5'-A(a) GCC TCG CTG CCG TCG CCA TAT CGT ACG GTT CTC

Sequence d5.

5'-AA (a)CC TCG CTG CCG TCG CCA TAT CGT ACG GTT CTC

Sequence d6.

5'-AA GC(a) TCG CTG CCG TCG CCA TAT CGT ACG GTT CTC

Sequence d7.

5'-AA GCC T(a)G CTG CCG TCG CCA TAT CGT ACG GTT CTC

Sequence d8.

5'-AA GCC TCG (a)TG CCG TCG CCA TAT CGT ACG GTT CTC

Sequence d9.

5'-AA GCC TCG CT(a) CCG TCG CCA TAT CGT ACG GTT CTC

Sequence d10.

5'-AA GCC TCG CTG C(a)G TCG CCA TAT CGT ACG GTT CTC

Sequence d11.

5'-AA GCC TCG CTG CCG (a)CG CCA TAT CGT ACG GTT CTC

Sequence d12.

5'-AA GCC TCG CTG CCG TC(a) CCA TAT CGT ACG GTT CTC

Sequence d13.

5'-AA GCC TCG CTG CCG TCG C(a)A TAT CGT ACG GTT CTC

Sequence d14.

5'-AA GCC TCG CTG CCG TCG CCA (a)AT CGT ACG GTT CTC

e) 3' Cy3 sequence

Sequence e1 (overhang).

5'-/biotin/TG GCG ACG GCA GCG AGG CTT AGC GGC AAA AAA AAA AAA AAA AAA AAA AAA AAA AAA AAG CCG CT(Cy3)

where (Cy3) is 3'-Cy3

Sequence e2 (nicked structure).

Top: 5'- AAG CCT CGC TGC CGT CGC CA

Bottom: 5'-/biotin/TG GCG ACG GCA GCG AGG CTT AGC GGC AAA AAA AAA AAA AAA AAA AAA AAA AAA AAA AAG CCG CT(Cy3)

where (Cy3) is 3'-Cy3

SUPPLEMENTARY FIGURES

a

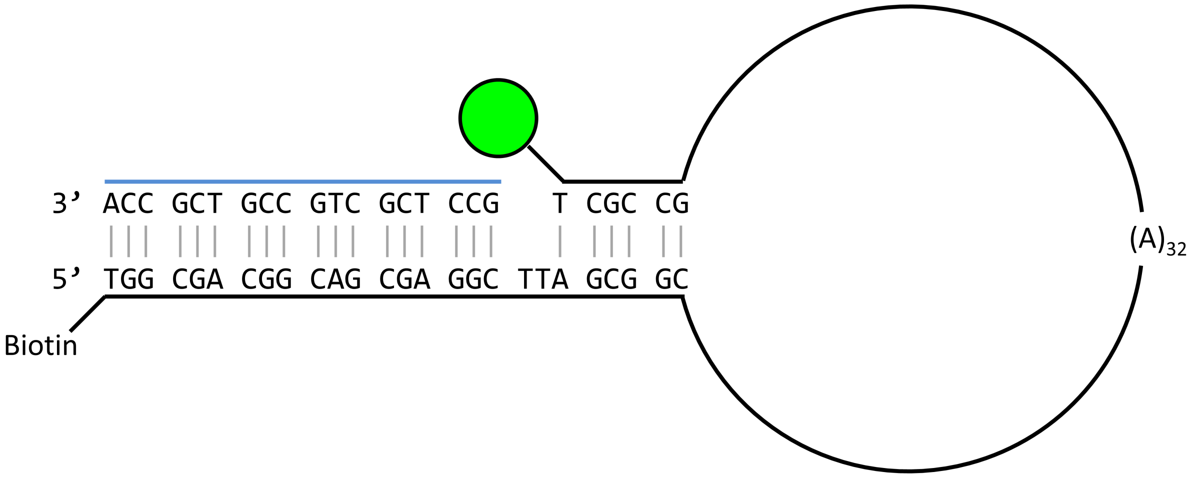

b

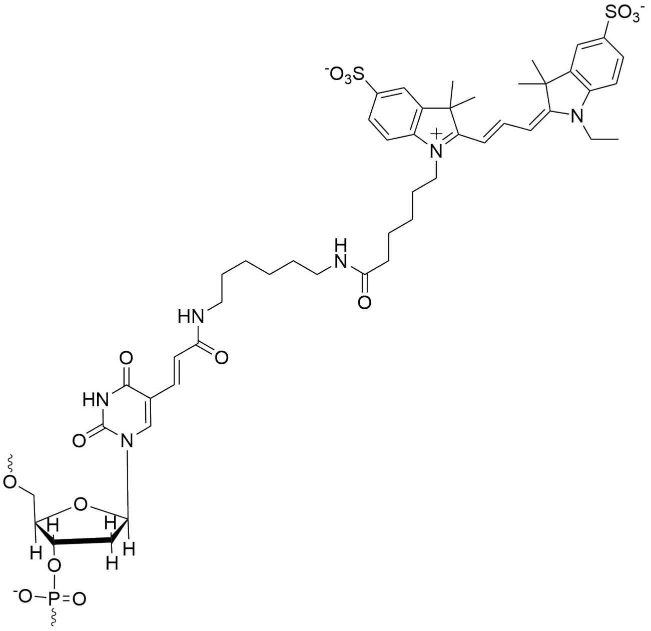

c

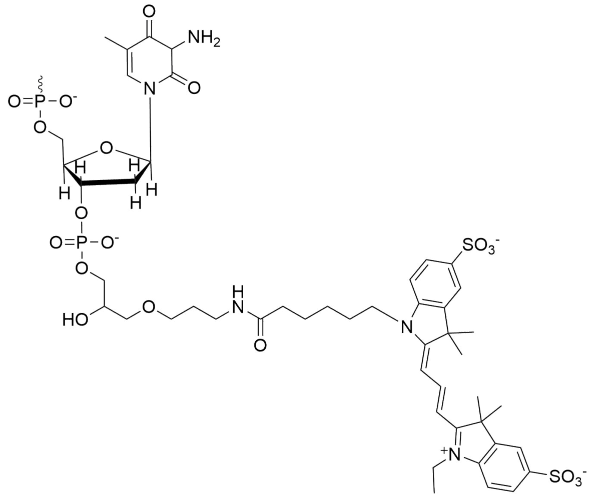

Supplementary Figure S1. (**a**) General structure of a fully-complementary hairpin, in this case with a 2 bp gap. (**b**) Structure of dT-Cy3. (c) Structure of 3'-Cy3.

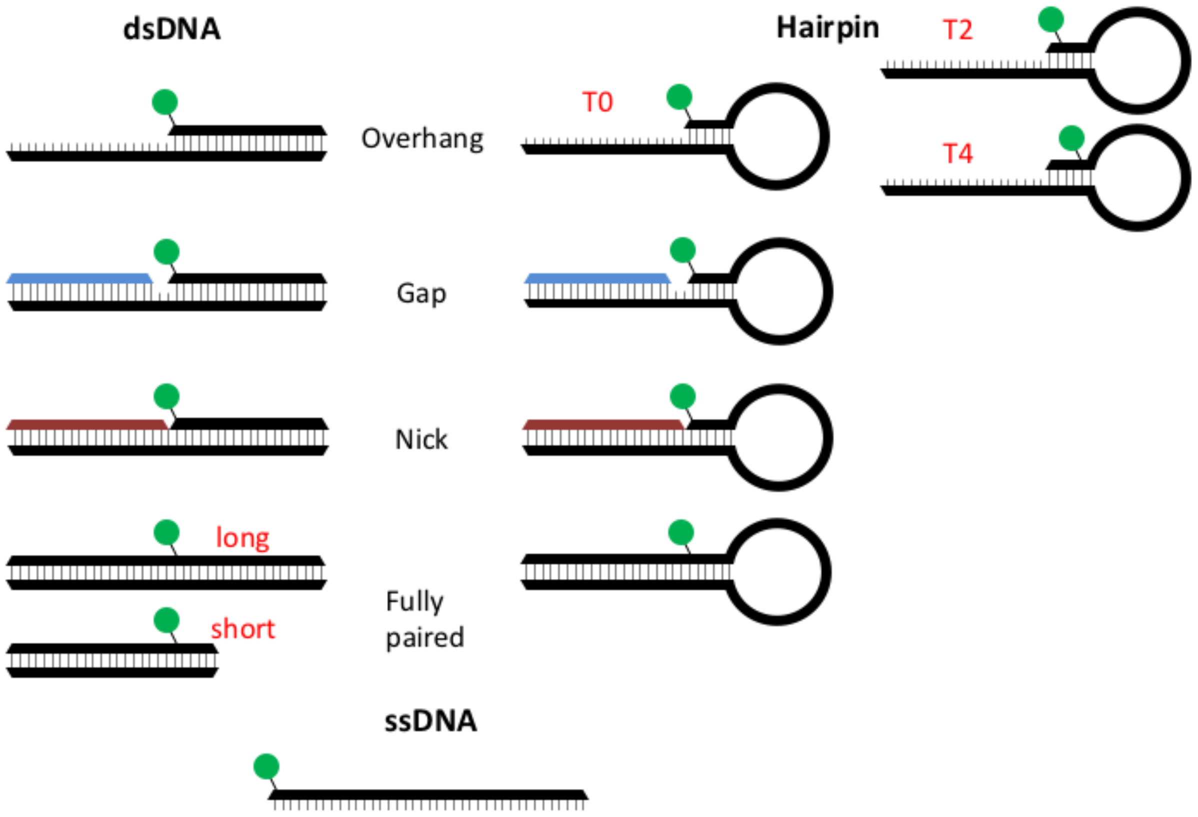

Supplementary Figure S2. Schematic of all DNA structures studied.

(a)

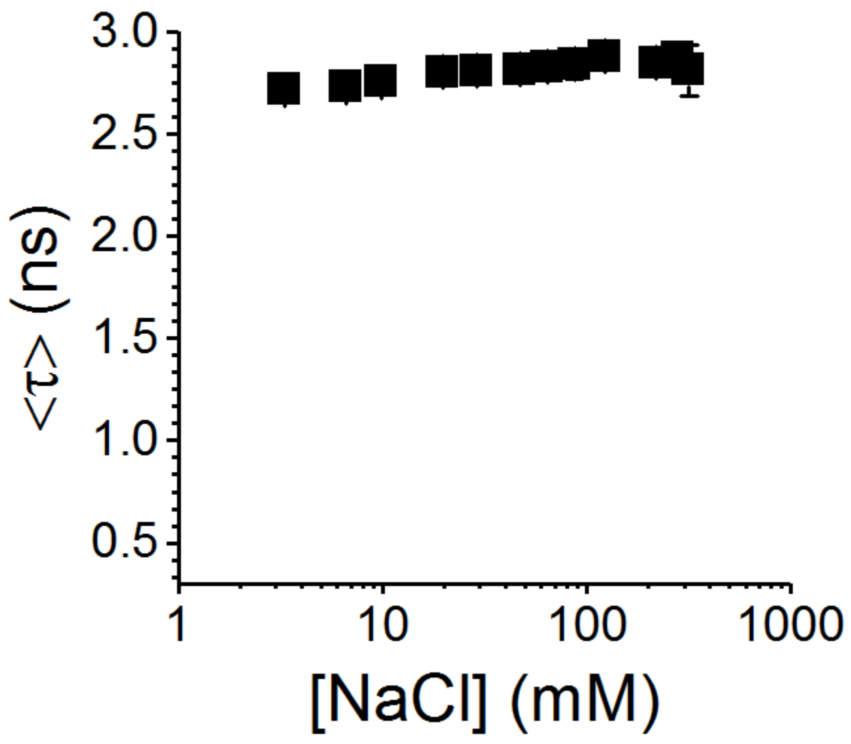

(b)

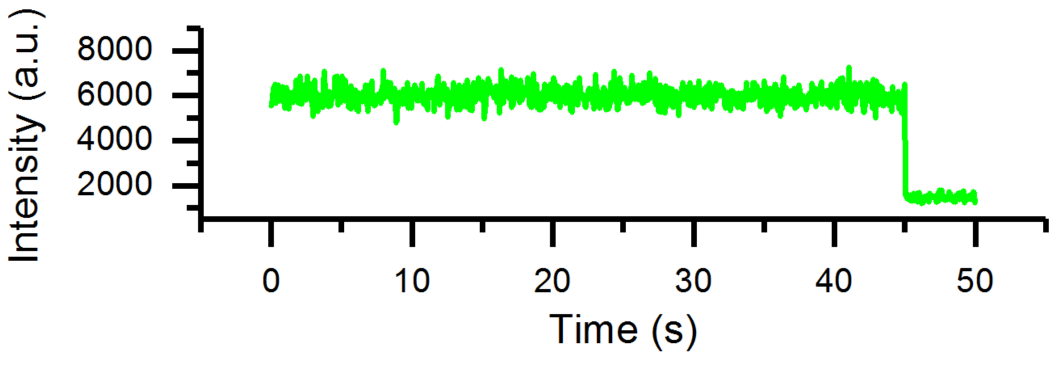

Supplementary Figure S3. (**a**) Amplitude-averaged lifetime of Cy3B hairpin (T0 overhang) vs. [NaCl]. (**b**) Representative single-molecule trace of Cy3B hairpin (T0 hairpin) in a buffer containing 10 mM NaCl.

a

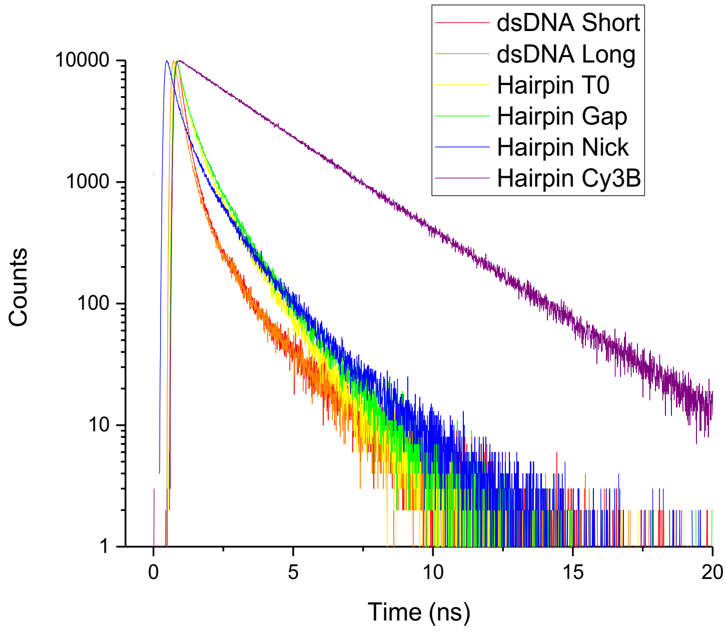

b

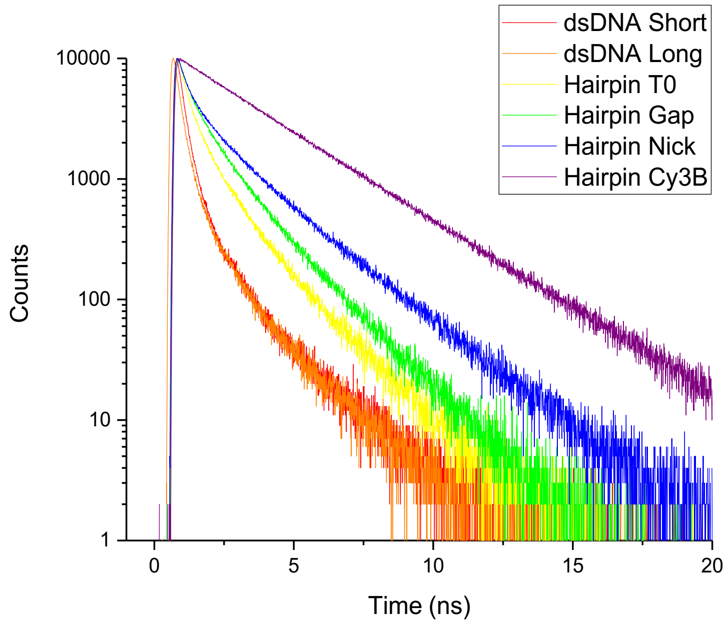

Supplementary Figure S4. TCSPC decays for duplexes and hairpins in buffer containing (**a**) 10 mM NaCl or (**b**) 50 mM NaCl.

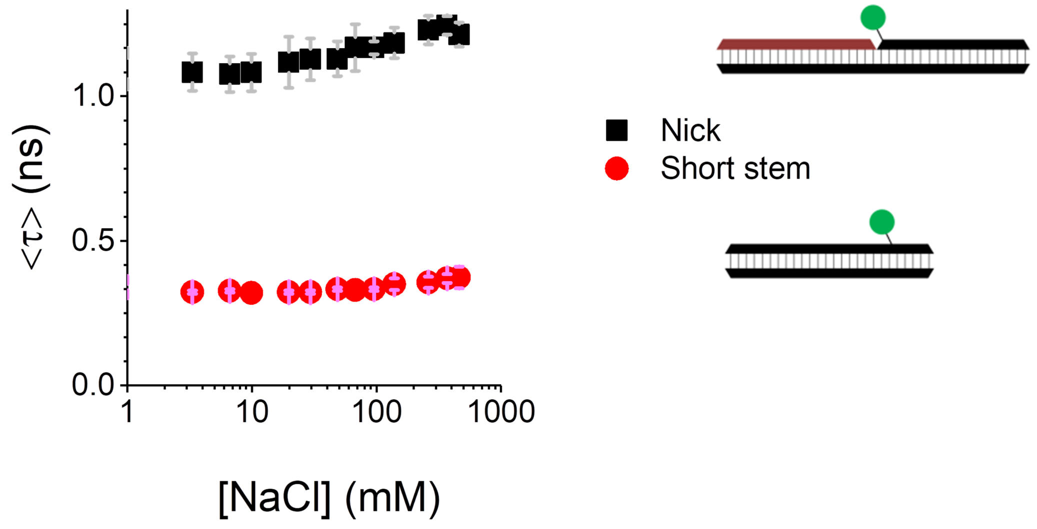

Supplementary Figure S5. Amplitude-averaged lifetime of dsDNA (nick and short stem duplexes) vs. [NaCl].

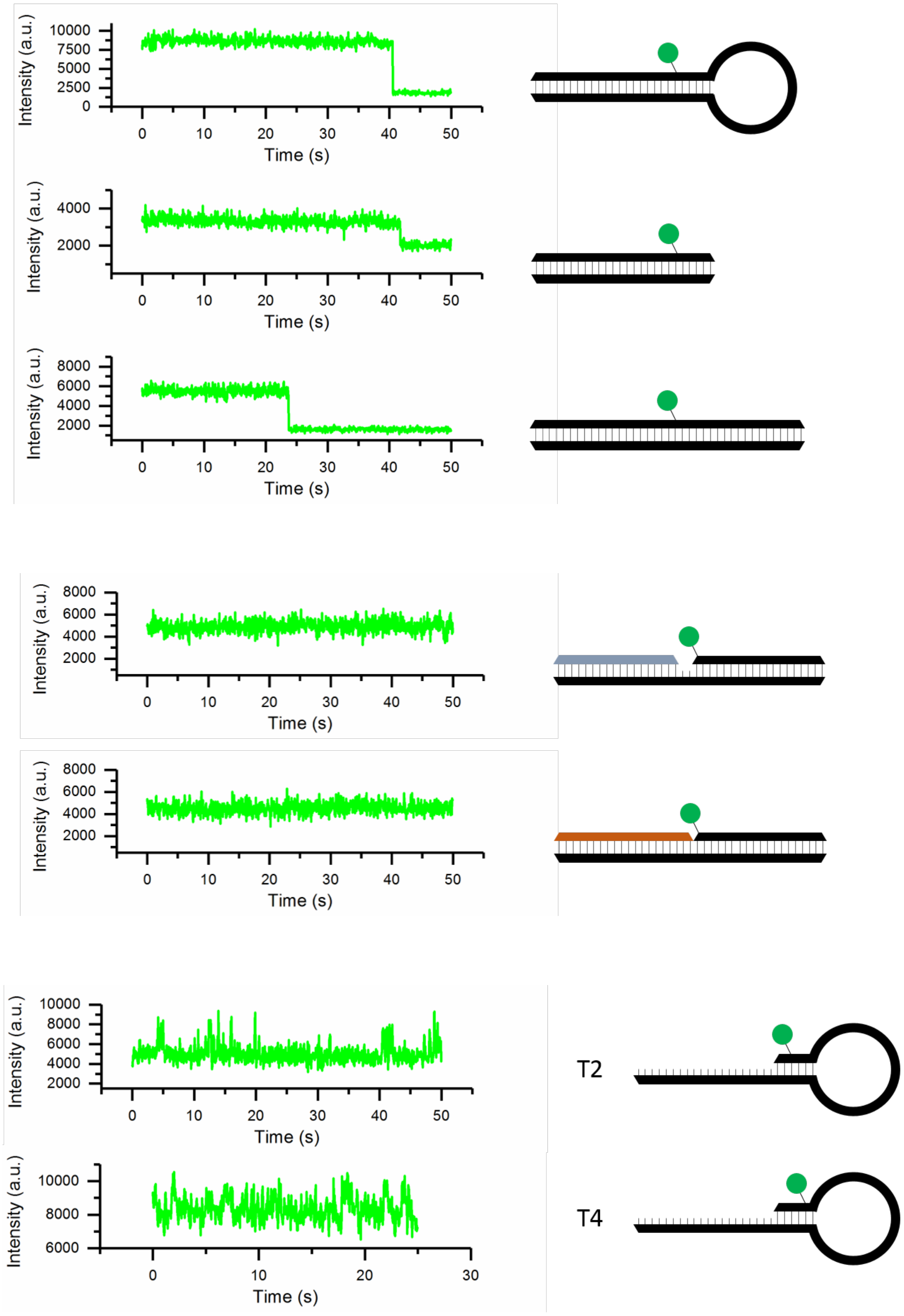

Supplementary Figure S6. Representative single-molecule traces of (top to bottom): the fully complementary hairpin, short duplex, long duplex, gapped duplex, nicked duplex, T2 hairpin and T4 hairpin (buffer contains 10 mM NaCl except for T2/T4 hairpins where there is 50 mM NaCl).

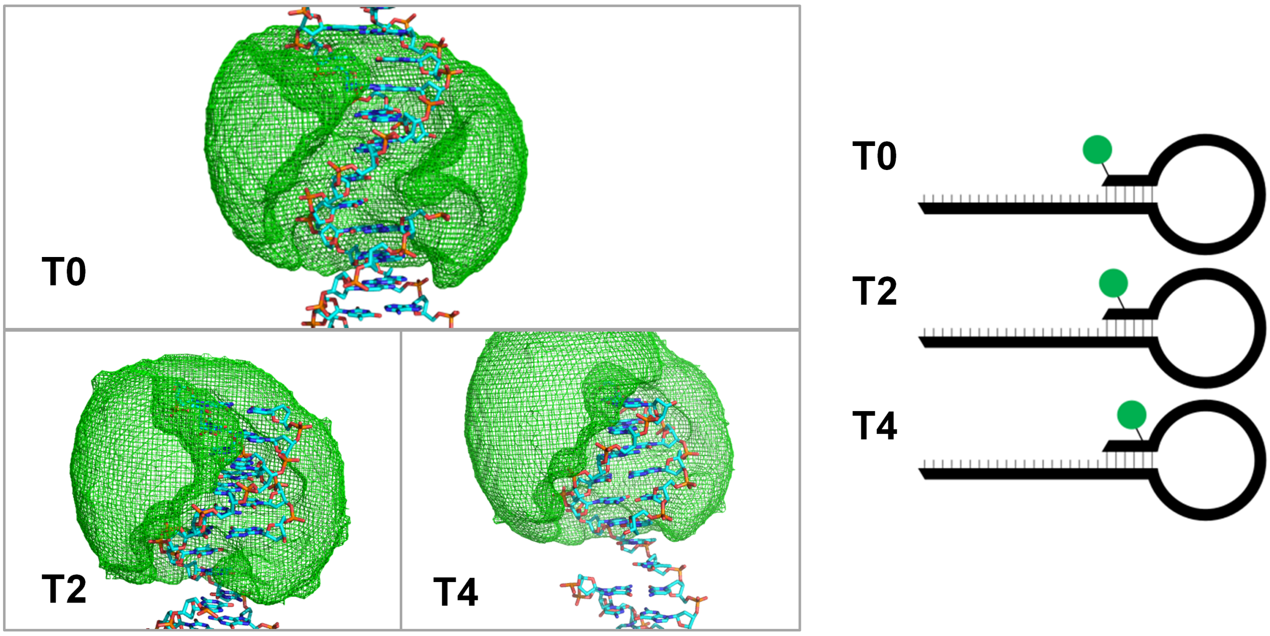

Supplementary Figure S7. AV simulations for T0, T2 and T4 with Cy3 internal dT linker (left). This is an enlarged version of panel (e) in Fig. 2 of the main text.

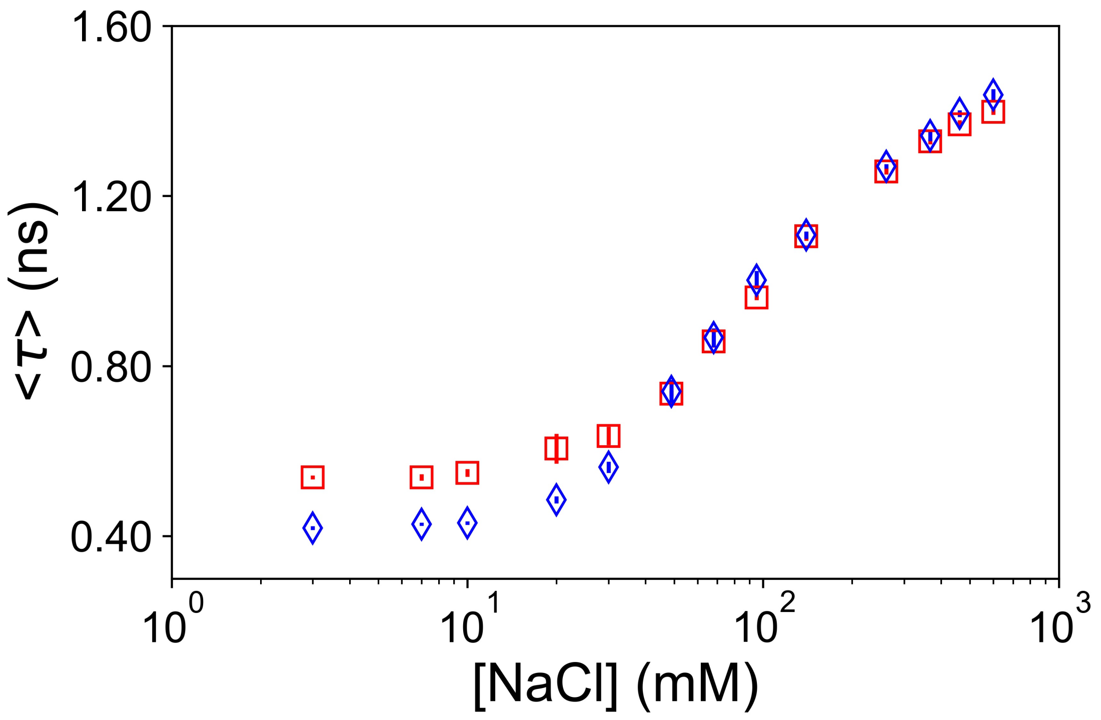

Supplementary Figure S8. Effect of dT vs 3′ labelling of Cy3 for overhang structure. Amplitude-averaged lifetime of the T0 overhang (dT-Cy3; squares) and the overhang formed by sequence e1 (3′-Cy3; diamonds). The error bars are the standard error of the mean (N = 3).

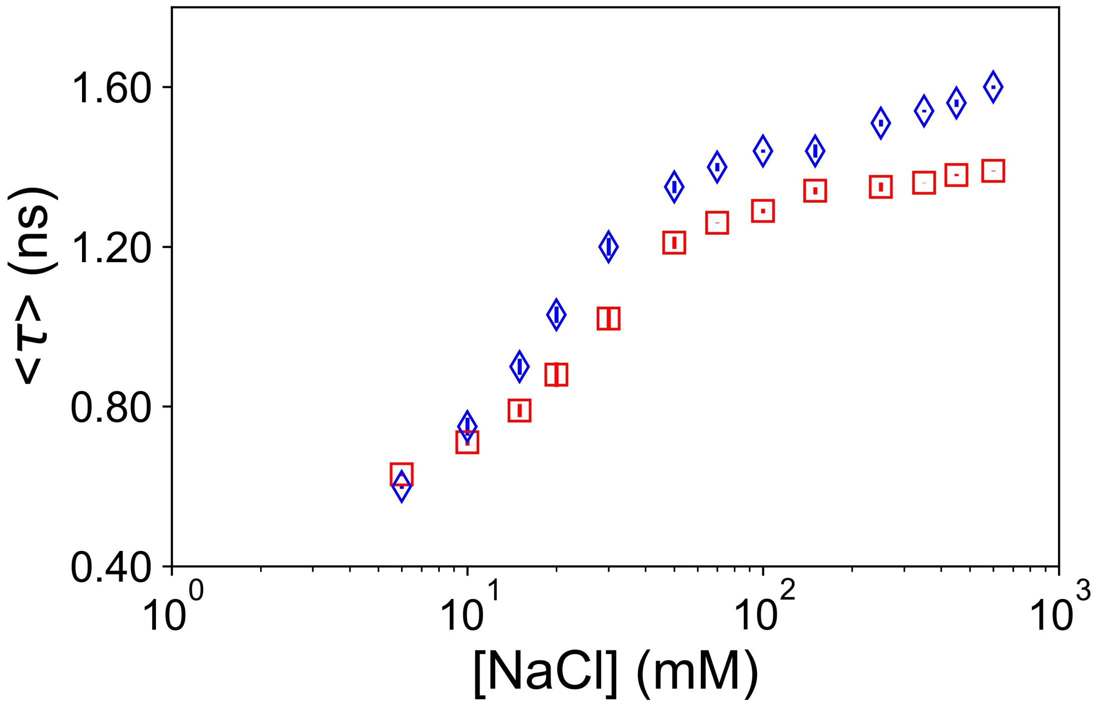

Supplementary Figure S9. Effect of dT vs 3′ labelling of Cy3 for nicked structure. Amplitude-averaged lifetimes of the nicked structure formed with sequence T0 (dT-Cy3; squares) and sequence e1 (3′-Cy3; diamonds). The error bars are the standard error of the mean (N = 3).

a

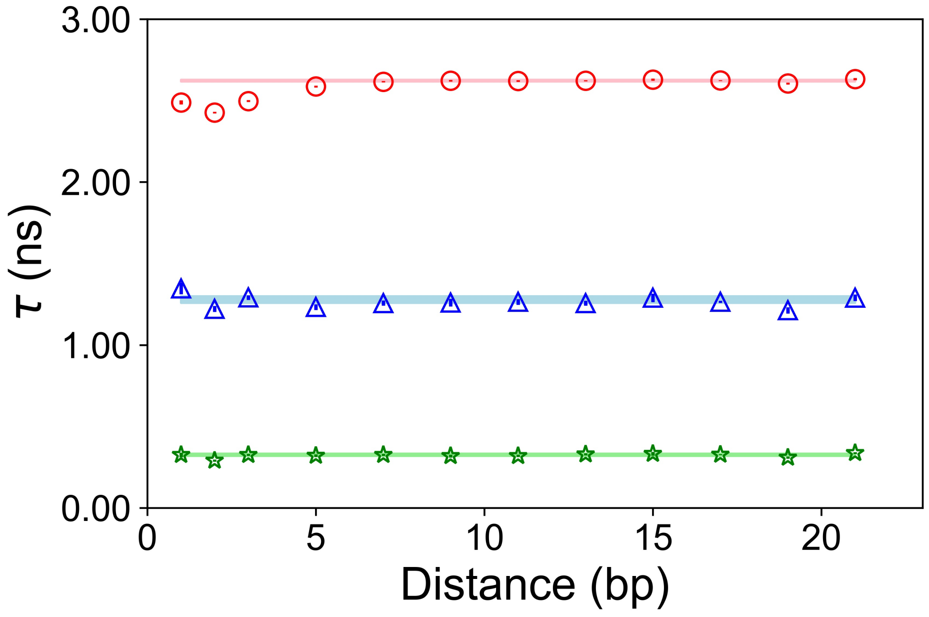

b

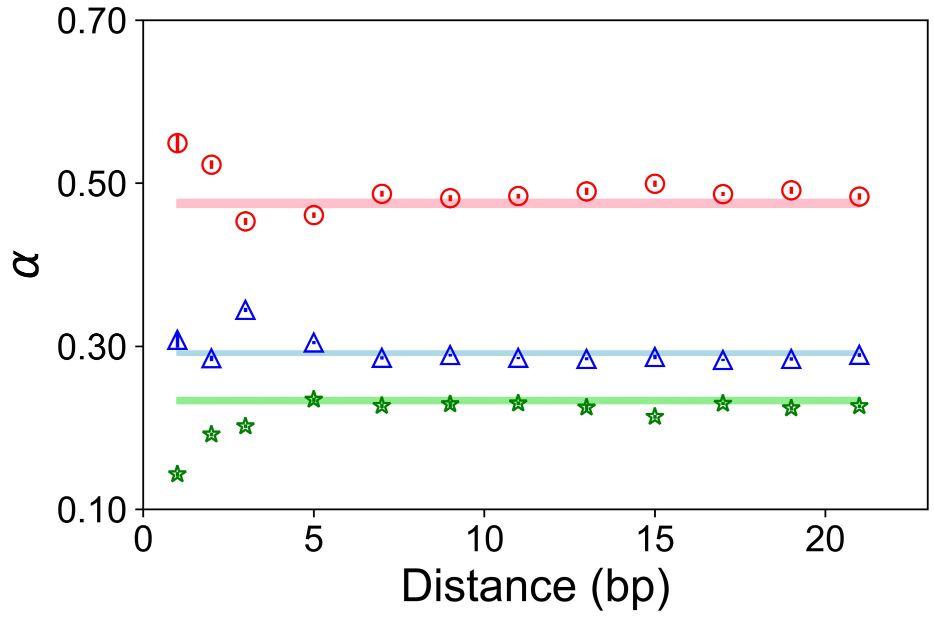

Supplementary Figure S10. Decay times (**a**) and amplitudes (**b**) from Table S3 (λ_{ex} = 532 nm, λ_{em} =565 nm, emission bandpass = 2.7 nm). The shaded bars correspond to the values obtained in the absence of abasic sites. In (**b**) the pre-exponential factors are shown for decay component 1 (≈ 2.6 ns, circles), 2 (≈ 1.3 ns, triangles), and 3 (≈ 0.3 ns, stars). Error bars are the standard error of the mean (N = 3). The fit parameters are shown in Supplementary Table S3.

Supplementary Tables

Supplementary Table S1. TCSPC data for hairpins. Buffer contained 50 mM NaCl.

Structure	τ_1 / ns	τ_2 / ns	τ_3 / ns	α_1	α_2	α_3	$<\tau>$ / ns	χ^2
T0	2.15 ± 0.02	0.87 ± 0.02	0.27 ± 0.01	0.14 ± <0.01	0.34 ± <0.01	0.52 ± <0.01	0.74 ± 0.01	0.91 ± 0.04
T2	1.58 ± 0.05	0.80 ± 0.05	0.25 ± 0.01	0.16 ± 0.02	0.42 ± <0.01	0.43 ± 0.02	0.69 ± 0.01	0.93 ± 0.01
T4	1.82 ± 0.04	0.74 ± 0.02	0.24 ± <0.01	0.11 ± 0.01	0.35 ± 0.01	0.54 ± 0.01	0.59 ± <0.01	0.93 ± 0.01
Cy3B	3.06 ± 0.02	1.07 ± 0.07		0.88 ± 0.01	0.12 ± 0.01		2.83 ± 0.01	1.01 ± 0.02
Full	2.09 ± 0.08	0.81 ± 0.09	0.24 ± 0.03	0.08 ± <0.01	0.20 ± 0.01	0.72 ± 0.02	0.50 ± 0.03	1.03 ± 0.04
Gap	1.81 ± 0.13	0.77 ± 0.14	0.21 ± 0.03	0.26 ± 0.03	0.38 ± 0.02	0.37 ± 0.05	0.84 ± 0.14	0.96 ± 0.04
Nick	2.47 ± 0.02	1.05 ± 0.04	0.28 ± 0.01	0.34 ± 0.02	0.25 ± 0.01	0.41 ± 0.02	1.21 ± 0.02	0.99 ± 0.02

All values are reported as the mean ± standard deviation for each parameter (τ_i, α_i, and $<\tau>$) calculated from three separate measurements.

Supplementary Table S2. TCSPC data for ssDNA and duplexes (overhang, gap, nick, short and long). Buffer contained 50 mM NaCl.

Structure	τ_1 / ns	τ_2 / ns	τ_3 / ns	α_1	α_2	α_3	$<\tau>$ / ns	χ^2
ssDNA	1.15 ± 0.07	0.47 ± 0.01	0.18 ± 0.01	0.08 ± 0.02	0.28 ± 0.02	0.64 ± 0.04	0.34 ± 0.01	0.98 ± 0.02
Overhang	1.90 ± 0.19	0.92 ± 0.09	0.29 ± 0.09	0.49 ± 0.03	0.39 ± 0.02	0.11 ± 0.02	0.92 ± 0.17	1.00 ± 0.06
Gap	2.04 ± 0.05	0.99 ± 0.03	0.28 ± 0.03	0.28 ± 0.02	0.41 ± 0.01	0.32 ± 0.03	1.06 ± 0.04	0.97 ± 0.05
Nick	2.40 ± 0.02	0.99 ± 0.04	0.27 ± 0.04	0.30 ± 0.02	0.32 ± 0.01	0.39 ± 0.01	1.13 ± 0.06	0.97 ± 0.03
Short	1.94 ± 0.40	0.64 ± <0.01	0.22 ± <0.01	0.11 ± 0.07	0.25 ± 0.06	0.65 ± 0.13	0.33 ± 0.01	1.00 ± 0.02
Long	1.89 ± 0.23	0.66 ± 0.01	0.22 ± 0.01	0.06 ± 0.04	0.18 ± 0.04	0.76 ± 0.09	0.32 ± 0.01	0.95 ± 0.02

All values are reported as the mean ± standard deviation for each parameter (τ_i, α_i, and $<\tau>$) calculated from three separate measurements.

Supplementary Table S3. TCSPC data for the hairpin sequences containing abasic sites. The buffer composition is 20 mM Tris, 600 mM NaCl (pH = 7.8).

Distance[a] / bp	τ_1 / ns	τ_2 / ns	τ_3 / ns	α_1	α_2	α_3	χ^2 range[b]
no abasic site	2.622 ± 0.005	1.279 ± 0.020	0.326 ± 0.006	0.475 ± 0.005	0.292 ± 0.002	0.233 ± 0.003	0.91-1.03
1	2.489 ± 0.013	1.348 ± 0.036	0.325 ± 0.015	0.549 ± 0.012	0.308 ± 0.009	0.143 ± 0.003	0.93-0.99
2	2.427 ± 0.005	1.222 ± 0.019	0.290 ± 0.007	0.523 ± 0.005	0.285 ± 0.003	0.192 ± 0.002	0.92-1.02
3	2.497 ± 0.006	1.292 ± 0.014	0.326 ± 0.007	0.453 ± 0.004	0.345 ± 0.003	0.202 ± 0.002	0.87-1.03
5	2.587 ± 0.006	1.233 ± 0.017	0.321 ± 0.008	0.461 ± 0.003	0.304 ± 0.002	0.235 ± 0.003	0.94-1.01
7	2.616 ± 0.005	1.258 ± 0.016	0.326 ± 0.006	0.487 ± 0.003	0.286 ± 0.002	0.227 ± 0.003	0.92-1.00
9	2.622 ± 0.006	1.260 ± 0.023	0.319 ± 0.009	0.482 ± 0.004	0.289 ± 0.002	0.229 ± 0.004	0.90-1.04
11	2.620 ± 0.005	1.265 ± 0.018	0.319 ± 0.008	0.484 ± 0.003	0.286 ± 0.002	0.230 ± 0.002	0.92-1.00
13	2.622 ± 0.004	1.257 ± 0.016	0.329 ± 0.006	0.490 ± 0.004	0.285 ± 0.002	0.225 ± 0.004	0.90-1.01
15	2.630 ± 0.007	1.290 ± 0.026	0.333 ± 0.012	0.500 ± 0.004	0.287 ± 0.003	0.214 ± 0.002	0.93-1.02
17	2.624 ± 0.003	1.265 ± 0.007	0.328 ± 0.003	0.487 ± 0.003	0.283 ± 0.001	0.230 ± 0.002	0.92-1.03
19	2.605 ± 0.006	1.213 ± 0.019	0.309 ± 0.006	0.491 ± 0.004	0.284 ± 0.002	0.224 ± 0.003	0.90-1.03
21	2.632 ± 0.007	1.290 ± 0.020	0.337 ± 0.006	0.484 ± 0.004	0.289 ± 0.002	0.227 ± 0.002	0.90-1.01

[a] Distance between the abasic site and the dT labeled with Cy3.
[b] The χ^2 range is for all of the individual decays.

Values are reported as the mean ± standard error calculated from thirteen decays (comprised of three or five repeats of three independent sample preparations).
